# Dysfunction of PTEN-Associated MicroRNA Regulation: Exploring Potential Pathological Links in Type 1 Diabetes Mellitus

**DOI:** 10.3390/medicina60111744

**Published:** 2024-10-24

**Authors:** Abdulhalim Senyigit, Sinem Durmus, Aykut Oruc, Remise Gelisgen, Hafize Uzun, Omur Tabak

**Affiliations:** 1 Department of Internal Medicine, Faculty of Medicine, Istanbul Atlas University, Istanbul 34403, Türkiye; abdulhalim.senyigit@atlas.edu.tr; 2 Department of Medical Biochemistry, Faculty of Medicine, İzmir Kâtip Celebi University, Izmir 35620, Türkiye; durmus.sinem@gmail.com; 3 Department of Physiology, Cerrahpasa Faculty of Medicine, Istanbul University-Cerrahpasa, Istanbul 34320, Türkiye; aykut.oruc@iuc.edu.tr; 4 Department of Medical Biochemistry, Cerrahpasa Faculty of Medicine, Istanbul University-Cerrahpasa, Istanbul 34320, Türkiye; remise.gelisgen@iuc.edu.tr; 5 Department of Medical Biochemistry, Faculty of Medicine, Istanbul Atlas University, Istanbul 34403, Türkiye; 6 Department of Internal Medicine, Kanuni Sultan Suleyman Training and Research Hospital, Health Sciences University, Istanbul 34668, Türkiye

**Keywords:** type 1 diabetes mellitus, microalbuminuria, miR-223, miR-106b, phosphatase and tensin homolog (PTEN)

## Abstract

*Background and Objectives:* Type 1 Diabetes Mellitus (T1DM) is an autoimmune disease with T cell-mediated pathogenesis of pancreatic β-cell destruction, leading to insulin deficiency. MicroRNAs such as miR-223 and miR-106b, along with PTEN, have been reported to participate in the pathophysiology of diabetes and its complications. The current study has explored the expression of miR-223, miR-106b, and PTEN and their association with various clinical and biochemical parameters in subjects diagnosed with T1DM. *Materials and Methods:* Sixty T1DM patients (two groups as uncomplicated/ with microalbuminuria) and fifty healthy volunteers, age- and sex-matched, were enrolled in this study. The fasting venous blood samples were collected, and PTEN and miRNAs (miR-223 and miR-106b) levels were measured by ELISA and real-time PCR, respectively. *Results:* The PTEN levels of patients with microalbuminuria were significantly lower than those of patients without microalbuminuria, while those of miR-223 and miR-106b were significantly increased in the T1DM group compared with the healthy control group (*p* < 0.001). ROC analysis indicated that PTEN, miR-223, and miR-106b could be potential biomarkers for diagnosing T1DM with high specificity but with variable sensitivities. Also, PTEN and miR-223 were negatively correlated with r =−0.398 and *p* < 0.0001, indicating that they were interrelated in their role within the T1DM pathophysiology. *Conclusions:* In the current study, it has been shown that the circulating levels of PTEN, miR-223, and miR-106b are significantly changed in T1DM patients and may back their potential to be used as non-invasive biomarkers for the diagnosis and monitoring of T1DM. Low PTEN protein expression was related to high miR-223 expression, indicating involvement of these miRNA in the regulation of PTEN. Further studies should be performed to clarify the exact mechanisms and possible clinical applications of these molecules.

## 1. Introduction

Type 1 Diabetes Mellitus (T1DM) is generally considered a T cell-mediated autoimmune disease against pancreatic β cells. This inflammatory process is further triggered by the involvement of inflammatory cells, cytokines, and chemokines. The pathogenesis of T1DM, mostly in genetically predisposed subjects, exhibits a complex interplay with immune dysfunction, viral infection, toxins, and dietary factors [1]. Specific destruction of the pancreatic β cells results in an insulin deficiency; current evidence strongly supports a role for cellular autoimmunity as a primary driving force in the development of disease [2].

MicroRNAs (miRNAs) are a class of noncoding RNAs of 18–24 nucleotides that have been shown to participate in the regulation of a wide range of biological processes [3]. They have been associated with the pathogenesis of chronic complications in diabetes (T1 and/or T2-DM) [4,5]. Although miRNAs have intracellular functions, the expression of miRNAs is detected in fluids of the organism, generally showing remarkable stability and usually expressing a specific profile of expression in some diseases [6,7,8]. Thus, circulating miRNAs could be offered as, at least in principle, potentially non-invasive clinical biomarkers for a wide range of pathophysiological states, including chronic complications of DM [9,10,11,12]. More specifically, in T1DM patients, miR-223 expression has been found to be significantly higher compared to healthy controls, associated with the development of diabetes, and acts as a response to lifestyle intervention [13,14].

Phosphatase and tensin homolog (PTEN), discovered in 1997, acts like a tumor suppressor. The PTEN gene is often inactivated in somatic cancers, and is probably the second most mutated tumor suppressor gene after *P53*. It is located at chromosome 10 and dephosphorylates phosphatidylinositol 3,4,5-trisphosphate, which in turn leads to the inactivation of Akt kinase [15]. PTEN negatively regulates protein kinase B (Akt) and the mammalian target of rapamycin (mTOR) pathways. An activated AKT/mTOR pathway, due to hyperglycemia, was cited as one of the reasons for the development of diabetic nephropathy. PTEN plays a role in renal fibrosis via mTOR [16].

Therefore, PTEN levels can modulate the activation status of Akt and thereby affect downstream signaling pathways that play a role in hypertrophy and matrix protein accumulation. Indeed, there have been reports of marked decreases in PTEN protein in a rat model of diabetic nephropathy and in cultured mesangial cells exposed to high levels of glucose. Indeed, these observations have recently been confirmed by other investigators [17]. However, precise mechanisms involved in high-glucose-mediated regulation of PTEN have yet to be clearly defined [18].

Our aim was conducted to determine the association of levels of miR-223, miR-106b, and PTEN with several clinical and biochemical parameters in T1DM-diagnosed individuals. Also, it is expected to add to the current state of knowledge concerning the roles of miR-223, miR-106b, and PTEN in the pathophysiology of T1DM, putting them further to light with regard to their effectiveness as non-invasive clinical biomarkers for the diagnosing and monitoring of diagnostic complications and, finally, perspectives on the management of the disease.

## 2. Materials and Methods

### 2.1. Subjects

This study was conducted according to the guidelines of the Declaration of Helsinki and approved by the Istanbul University-Cerrahpasa, Cerrahpasa Medical Faculty Clinical Research Ethics Committee (number of approval: 1061259; Date: 9 August 2024). All subjects gave their informed consent for inclusion before they participated in the study. All subjects were of Turkish descent.

### 2.2. Study Groups

1. Control group: Fifty healthy volunteers who were age- and gender-matched with the diabetic group. Volunteers who came to the hospital for a check-up, without any disease, and had fasting plasma glucose levels < 100 mg/dL and plasma glucose levels < 140 mg/dL at 2 h after oral glucose tolerance testing (OGTT) were included in the healthy group.
2. Patients group: The study included 60 patients diagnosed with T1DM who presented to the diabetes outpatient clinics of Istanbul Atlas University, Medicine Hospital. The diagnosis of T1DM was made according to the diagnostic criteria set out in the guidelines of the American Diabetes Association [19]. Patient groups were divided into 2 groups as either uncomplicated and with microalbuminuria.

Detection of antibodies against glutamic acid decarboxylase antibody (GADA), autoantibodies against insulin (IAA), and islet cell autoantibodies (ICA) is valuable in the diagnosis of immune-induced T1DM. Since GADA, IAA, and ICA could not be studied at the time the patients were diagnosed and some patients were not recorded in their files, only the patients for which information was available were evaluated. Antibody tests could not be performed in the control groups. The information of patients who underwent diabetes-specific autoantibody tests outside our hospital was obtained from the patient file.

Insulin regimens used by all patients and daily dose (units/kg) were recorded.

### 2.3. Inclusion Criteria

The patient group (T1DM) consisted of individuals with diabetes who were diagnosed before age 40 years, treatment with insulin, current age 20–50 years, and diabetes duration 3–25 years.

### 2.4. Exlusion Criteria

All participants in the study were screened for hypertension, hyperlipidemia, chronic obstructive pulmonary disease (COPD), coronary artery disease (CAD), peripheral artery disease (PAD), and cardiac and cerebrovascular diseases and symptoms (such as angina, history of myocardial infarction, history of stroke, heart failure, documented diseases related to aortic, coronary, and peripheral artery disease, and previous surgeries). İndividuals with these conditions were excluded in the study. Patients with autoimmune diseases such as Multiple Sclerosis (MS), intestinal diseases such as ulcerative colitis, Crohn’s disease and celiac disease, skin diseases such as psoriasis, pemphigus vulgaris, and vitiligo, as well as Graves’ disease, Hashimoto’s disease, and rheumatoid arthritis were excluded. Patients with a history of uveitis or glaucoma were also excluded.

### 2.5. Sample Collection and Measurements

Fasting venous blood samples were drawn between 8 and 10 am after the subjects fasted overnight (10–12 h). Blood samples were drawn from the brachial veins in brachial fossa and placed into plain tubes and anticoagulant-free tubes. The samples were centrifuged for 10 min at 4000 rpm at 4 °C. Biochemical tests were performed immediately. For the determination of other parameters, serum aliquots were frozen and stored at −80 °C immediately until they were required for further analysis.

Participants’ height, weight, body mass index (BMI), and duration of diabetes were obtained from their medical records.

Brachial arterial pressure was obtained by a single doctor using a mercury sphygmomanometer (Big Ben^®^ round; Riester, Jungingen, Germany) standardized in accordance with the American and British Hypertension Society and the World Health Organization recommendations.

Evaluation of diabetic nephropathy was performed by calculating the glomerular filtration rate (GFR). The GFR was calculated by the Cocroft-Gault (CG) formula using gender and body surface area [20].

GFR (MDRD) = 175 × standardized S_cr_^−1.154^ × age^−0.203^ × 1.212 [if black] × 0.742 [if female]

Microalbuminuria was determined from three separate 24 h urine samples collected at least one month apart by a measured nephelometric method. Excretion of 30–300 mg/day albumin in urine in at least two of three samples was considered microalbuminuria.

Biochemical parameters were determined using the enzymatic methods (Architect i2000, Abbott Park, IL, USA). Insulin levels were measured by the electrochemiluminescence immunoassay (ECLIA) method on Roche-Hitachi E170 (Roche/Hitachi MODULAR Analytics Combination Systems, Roche Diagnostics, Indianapolis, IN, USA). HbA1c determination was based on HPLC (Variant Turbo II, Bio-Rad Laboratories, Inc., Hercules, CA, USA). Homeostasis model assessment for insulin resistance (HOMA-IR) was calculated by using the following formula:

HOMA-IR = Fasting glucose (mg/dL) × Fasting insulin (mU/L)/405

### 2.6. Measurement of Serum Phosphatase and Tensin Homolog (PTEN) Levels

Serum PTEN levels were determined by using a commercially available human enzyme-linked immunosorbent assay (ELISA) kit according to the manufacturer’s instructions (MyBioSource, Cat. No: MBS2705917, MyBioSource, Inc., San Diego, CA, USA). All samples were examined twice. The minimum measurable level for serum asprosin was 0.775 ng/mL. The intra- and inter-CV for asprosin were determined to be 10% and 12%, respectively.

### 2.7. Analysis of MicroRNA Expression Levels

miRNAs were extracted from venous blood samples using an A.B.T.™ miRNA Purification Kit (Atlas Biotechnology, Ankara/Türkiye). Quantification of extracted RNA were realized by using a Qubit™ microRNA Assay Kit (Life Technologies, Carlsbad, CA, USA) with the Qubit^®^ 2.0 Fluorometer (Life Technologies, Carlsbad, CA, USA). At the first step, miRNA samples were transcribed into complementary DNA (cDNA) using the A.B.T.™ cDNA Synthesis Kit with Rnase inh. (High Capacity) (Atlas Biotechnology, Ankara/Türkiye) for estimating the expression rate. Then, miRNA expression levels were analyzed with MicroRNA Assays (hsa-miR-223, hsa-miR-106b and U6; Atlas Biotechnolog) using the StepOnePlus real-time PCR system (Applied Biosystems, Carlsbad, CA, USA). The relative expression levels of miR-223 and miR-106b were calculated using the 2^−ΔΔCT^ method.

### 2.8. Statistical Analysis

In this study, we performed a comprehensive statistical analysis to compare the levels of various biomarkers between the diabetic and control groups. Descriptive statistics, including means and standard deviations, were calculated for all numeric variables. To assess the differences in means between the groups, independent *t*-tests were conducted, and the significance of these differences was determined using *p*-values, with a threshold of *p* < 0.05 being considered to be statistically significant. Additionally, fold change calculations were performed using the 2^−ΔΔCt^ method for miR223 and miR106b, comparing their expression levels between the two groups. The relative increase in expression was quantified and the significance of these differences was tested. For the ROC (Receiver Operating Characteristic) curve analysis, PTEN levels were evaluated to assess their discriminatory power between the diabetic and control groups. Given that lower PTEN levels are associated with the diabetic group, the PTEN values were inverted (i.e., subtracted from a constant) to better align with the positive diagnostic direction of the ROC curve analysis. This inversion was necessary to ensure that higher values corresponded with the presence of diabetes, thereby improving interpretability. The area under the curve (AUC) was calculated to determine the diagnostic accuracy of PTEN and other biomarkers. Univariate logistic regression analyses were performed to determine associations between each individual marker and microalbuminuria.

## 3. Results

In this study, the mean age of the participants did not significantly differ across groups, with controls at 37.78 ± 8.37 years, T1DM without complications at 36.37 ± 8.05 years, and DM with microalbuminuria at 39.73 ± 14.39 years (Table 1).

The gender distribution was similar across the three groups. In the control group, there were 31 females and 19 males. The T1DM with microalbuminuria group consisted of 16 females and 14 males, while the DM without complications group had an equal distribution of 15 females and 15 males. A chi-square test indicated no statistically significant difference in gender distribution among the groups (*p* = 0.535), suggesting comparable gender representation in each group. The mean duration of diabetes was 8.2 ± 5.09 years in the DM without complications group and 8.9 ± 7.33 years in the T1DM with microalbuminuria group. There was no statistically significant difference in diabetes duration between the two groups (*p* = 0.669). Most of our patients received intensive insulin treatment. There was no statistically significant relationship between insulin doses and the groups. The BMI was significantly higher in both T1DM groups compared to controls (27.55 ± 3.02 kg/m² in DM without complications, 32.38 ± 6.84 kg/m² in DM with microalbuminuria, and 24.58 ± 0.8 kg/m² in controls, *p* < 0.001). In the DM with microalbuminuria group, there were 18 individuals who were classified as patients with obesity (BMI ≥ 30 kg/m²). Similarly, waist circumference was significantly higher in both DM groups compared to controls (*p* < 0.001). Systolic and diastolic blood pressure values were significantly higher in the DM with microalbuminuria group compared to both controls and the DM without complications group (*p* < 0.001). Fasting glucose and HbA1c levels were significantly elevated in both DM groups compared to controls (*p* < 0.001), with the DM with microalbuminuria group showing even higher HbA1c levels than the DM without complications group (*p* < 0.001). Insulin and HOMA-IR levels were also significantly elevated in both DM groups compared to controls and higher in the DM with microalbuminuria group compared to the DM without complications group (*p* < 0.001). Notably, microalbuminuria levels were markedly higher in the DM with microalbuminuria group (193.56 ± 247.29 mg/L) compared to both controls (4.95 ± 3.66 mg/L) and the DM without complications group (4.32 ± 0.29 mg/L, *p* < 0.001).

Total cholesterol levels were significantly higher in the DM without complications group compared to controls (*p* < 0.01), while triglyceride and VLDL levels were elevated in both DM groups compared to the controls (*p* < 0.01). HDL levels were significantly lower in the DM with microalbuminuria group compared to the controls (*p* < 0.01). However, there was no significant difference in LDL levels among the groups. These results demonstrate that the DM groups, particularly those with microalbuminuria, exhibited significant metabolic and cardiovascular risk factors compared to controls.

**Figure 1:**
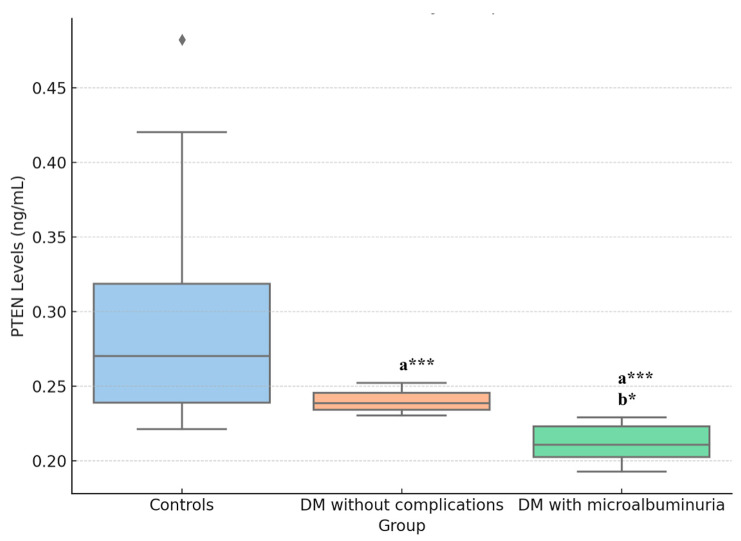
Comparison of PTEN levels. **a**: means compared to controls; **b**: means compared to DM without complications. *: *p* < 0.05; ***: *p* < 0.001. PTEN levels were measured in 50 control subjects, 30 DM patients without complications, and 30 DM patients with microalbuminuria by ELISA analysis.

**Figure 2:**
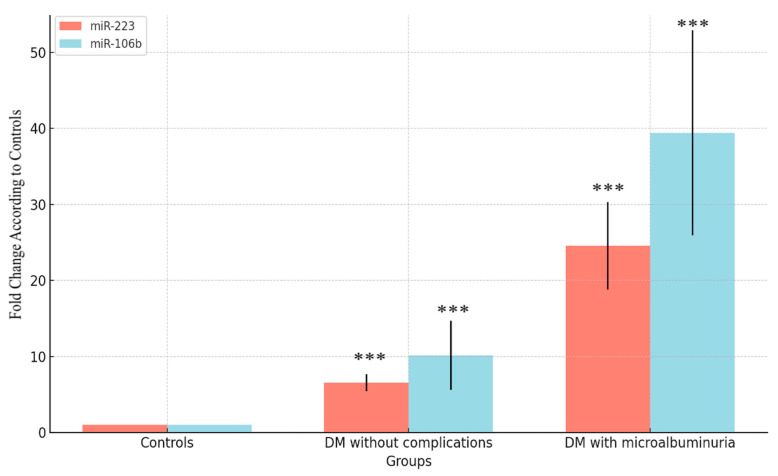
Fold change comparisons for miR223 and miR106b vs. control group. Expression analyses were performed in 50 control subjects, 30 DM patients without complications subjects, and 30 DM patients with microalbuminuria. In the fold change calculation, control individuals were used as reference and fold change was calculated compared to control individuals. Fold change calculations were performed using the 2^−ΔΔCt^ method, and the significance of differences in this relative expression was tested by using ANOVA and a post hoc Tukey test. *** means *p* < 0.001.

PTEN levels were significantly lower in both DM with microalbuminuria and DM without complications groups compared to the control group (*p* < 0.001) (Figure 1). Additionally, PTEN levels in the DM with microalbuminuria group were significantly lower than in the DM without complications group (*p* = 0.024, Figure 2). The negative correlation between PTEN with fasting glucose (r = −0.784, *p* < 0.001) and duration of diabetes (r = −0.592, *p* < 0.001) is statistically significant. The negative correlation between PTEN and miR223 is statistically significant (r = −0.398, *p* < 0.0001), suggesting a meaningful relationship between these two variables. The correlation between PTEN and miR106b (r = −0.170, *p* = 0.0903) however, is not statistically significant, indicating that the observed relationship may be due to chance.

Autoantibody data of the patients are given in Table 2.

The ratios of PTEN levels to microalbuminuria, HbA1c, insulin, and HOMA-IR were significantly different across the groups (Table 3). The PTEN/microalbuminuria ratio was progressively lower in the control group (0.099 ± 0.076) than in the DM without complications (0.055 ± 0.005) and DM with microalbuminuria (0.003 ± 0.008) groups, with all comparisons being highly significant (*p* < 0.001). The PTEN/HbA1c ratio was significantly reduced in the DM groups compared to controls (*p* < 0.001), but no significant difference was found between the DM groups (*p* = 1.000). Similarly, the PTEN/insulin ratio showed a significant reduction in the DM groups (*p* < 0.001), although the difference between DM groups was not significant (*p* = 0.157). Lastly, the PTEN/HOMA-IR ratio was significantly lower in the DM groups compared to controls (*p* < 0.001), with no significant difference being seen between the DM subgroups (*p* = 0.066).

Table 4 shows the comparison of various models examining the relationship between DM with the microalbuminuria group and uncomplicated DM in logistic regression models. Different combinations of PTEN, miR223, and miR106b biomarkers were evaluated according to the cut-off values obtained because of ROC analysis. Probability (P(M)), conditional probability with data (P(M|data)), the Bayes Factor Model (BFM), comparison with the best model (BF10), and R² values are presented for each model. The model with the highest P(M|data) value and the strongest Bayes factor is the model including the combination of PTEN > −0.23, miR223 > 6.19 and miR106b > 154.25 (BFM = 1.753, BF10 = 1.000). Other models have lower probabilities and Bayes factors compared to this model. Models using miRNAs alone showed poor performance, while BFM and BF10 values remained quite low in models using miR106b and miR223 separately or in combination. These results show that using PTEN and two miRNAs together is the most effective model in determining microalbuminuria.

The fold change in miR-223 and miR-106b was significantly different across the groups. For miR-223, the fold change was significantly higher in the DM with microalbuminuria group (*p* < 0.001). ANOVA analysis confirmed a highly significant difference in miR-223 expression between the groups (*p* < 0.001). Similarly, miR-106b expression showed a significant increase in the DM with microalbuminuria group compared to controls (*p* < 0.001), with ANOVA indicating a statistically significant difference across the groups (*p* = 0.00034). These results suggest a notable upregulation of miR-223 and miR-106b in diabetic patients with microalbuminuria (Figure 2).

**Figure 3:**
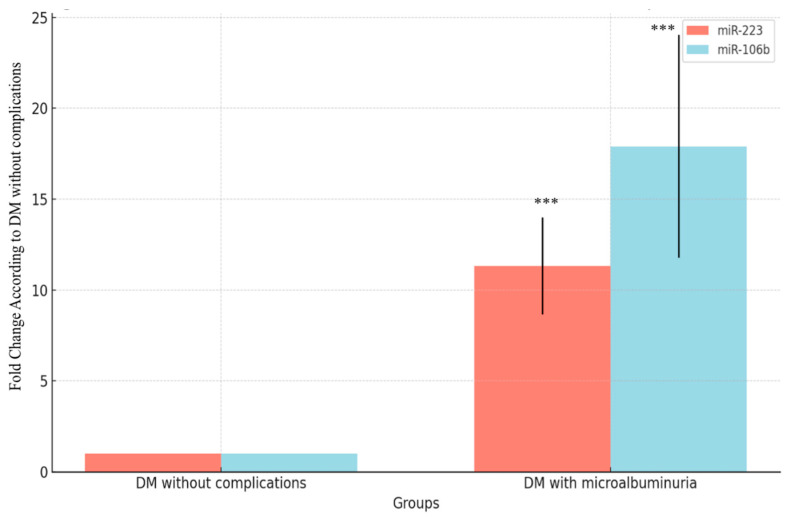
Fold change comparisons for miR223 and miR106b vs. DM without complications. Expression analyses were performed in 30 DM patients without complications and 30 DM patients with microalbuminuria. In the fold change calculation, 30 DM patients without complications were used as references and fold change was calculated compared to DM patients without complications. Fold change calculations were performed using the 2^−ΔΔCt^ method, and the significance of differences in this relative expression was tested by using ANOVA and a post hoc Tukey test. *** means *p* < 0.001.

When using the DM without complications group as the reference (Figure 3), the fold change for miR-223 was significantly higher in the DM with microalbuminuria group, with a fold change of 11.33 (*p* < 0.001). Similarly, for miR-106b, the fold change in the DM with microalbuminuria group was 17.91 compared to the DM without complications group (*p* < 0.001). Both miR-223 and miR-106b showed substantial upregulation in the DM with microalbuminuria group, indicating a strong association between miRNA expression and the presence of microalbuminuria in diabetic patients.

**Figure 4:**
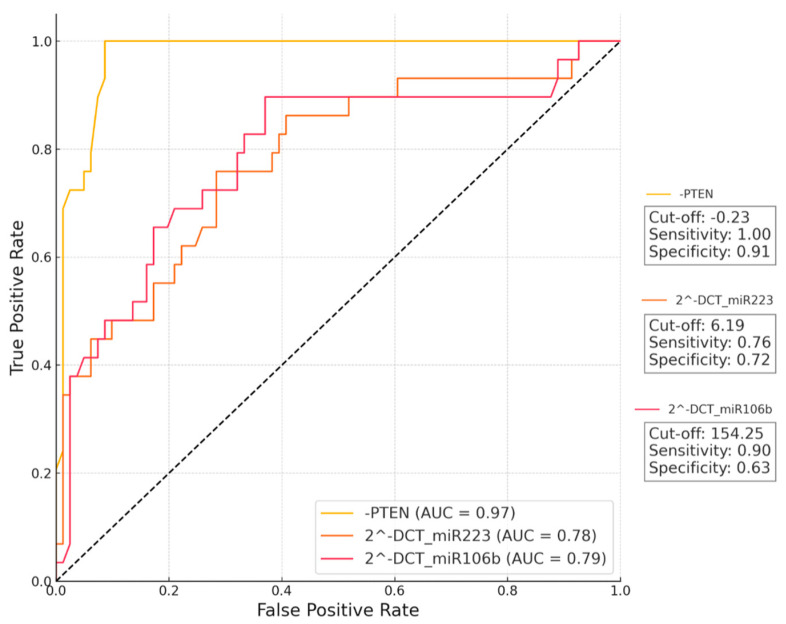
ROC curves for PTEN, miR223, and miR106b for DM without complications vs. DM with microalbuminuria. ROC analyses were performed in 30 DM patients without complications and 30 DM patients with microalbuminuria. AUC values, sensitivity values, and specificity values for each parameter are indicated in the boxes in the figure. -PTEN means that, given that lower PTEN levels are associated with the diabetic group, the PTEN values were inverted (i.e., subtracted from a constant) to better align with the positive diagnostic direction of the ROC curve analysis. This inversion was necessary to ensure that higher values corresponded with the presence of diabetes, thereby improving interpretability.

PTEN demonstrated an excellent discriminative ability between the two groups, with an AUC of 0.97, indicating near-perfect performance (Figure 4).

The optimal cut-off value of −0.23 achieved 100% sensitivity and 91% specificity, making PTEN a highly reliable marker for detecting microalbuminuria in diabetic patients. In contrast, miR223 and miR106b showed a moderate diagnostic power, with AUC values of 0.78 and 0.79, respectively. For miR223, a cut-off value of 6.19 resulted in 75.86% sensitivity and 71.60% specificity, while miR106b had a cut-off value of 154.25, achieving 89.66% sensitivity and 62.96% specificity.

## 4. Discussion

The pathogenesis of T1DM is not fully understood. Despite the utility of the autoantibodies in T1DM prediction, they have several limitations [21,22]. Therefore, new biomarkers of T1DM are needed to complement the information obtained from the presence of autoantibodies together with other risk factors. PTEN levels were found to be significantly lower in T1DM compared to the control group, indicating a notable reduction in PTEN among diabetic patients. Microalbuminuria is non-invasive biomarker and the earliest sign of diabetic nephropathy in T1DM patients. PTEN levels of patients with microalbuminuria were significantly lower than those of patients without microalbuminuria. Both miR223 and miR106b show a significant increase in expression in the diabetic group compared to the control group. A negative correlation was found between PTEN and miR223. Our study is the first to show that PTEN levels are significantly reduced in patients with microalbuminuria in T1DM. Low PTEN protein expression was related to high miR-223 expression, indicating the involvement of these miRNA in the regulation of PTEN.

Cardiovascular and renal complications share common risk factors such as blood pressure, blood lipids, and glycemic control. Thus, chronic kidney disease can predict cardiovascular disease in the general population [23]. In the current study, HbA1c levels were significantly higher in the diabetic patients versus the control group, reflecting poor glycemic control in T1DM. Similarly, FBG was markedly elevated in the diabetic group compared to the control group. The HbA1c levels of patients with microalbuminuria were significantly higher than those of patients without microalbuminuria. Poor metabolic control and related high HbA1c levels in T1DM patients were found to be one of the main determinant factors in our study, in accordance with the literature [24]. All patients with systolic HT and microalbuminuria had high HbA1c levels. In terms of microvascular complications, duration of disease and high systolic blood pressure (SBP) values were found to be risk factors for diabetic retinopathy in both T1DM and T2DM patients [25]. Another study showed that patients with microalbuminuria and high SBP had significant left ventricular dysfunction [26]. In our current study, only 18 of the patients with microalbuminuria were patients with obesity. Similarly, waist circumference was significantly higher in both DM groups compared to controls. Studies have found a relationship between obesity and microalbuminuria [27]. In patients with T1DM where the onset of diabetes can be exactly defined, the onset of microalbuminuria and then macroalbuminuria closely correlates with the duration of diabetes [28]. Strict glycemic control is the main preventive factor of diabetic nephropathy in T1DM.

Autoantibodies are not detectable at any stage in some individuals, which complicates diagnosis of T1DM [29], particularly in adults. Since GADA, IAA, and ICA could not be studied at the time of diagnosis and were not recorded in the files of some patients, only the patients whose data were obtained were evaluated. In our current study, GAD65 was found to be positive in 82.6% of 25 patients, IAA in 52.5% of 35 patients, and ICA in 60.3% of 22 patients (Table 2). Autoantibodies act as reliable markers of T1DM susceptibility and help to categorize cohorts that may benefit from intervention. The US Food and Drug Administration (FDA) in 2022 approved the drug teplizumab, an anti-CD3 monoclonal antibody, the first treatment aimed at delaying the onset of T1DM, for use in people aged eight years and older. Clinical use of teplizumab is currently limited to relatives of individuals with T1DM with dysglycemia who have two or more islet autoantibodies but have not yet reached the overt diabetes stage. In individuals with recently diagnosed T1DM, administration of this treatment has been shown to protect β-cells but not lead to an improvement in HbA1c [30]. The risks of immunosuppression, side effects, potential long-term safety issues, variable efficacy, high cost, and the need for regular monitoring must be carefully considered when deciding on its use [31].

The pathogenesis of diabetic nephropathy is multifactorial. Hyperglycemia has a direct effect on glomerular, vascular, tubular, and interstitial renal cells [30]. PTEN is dynamically regulated in kidney injury, suggesting that PTEN may harbor post-translational modifications which play important roles in kidney disease. Some studies have reported that single nucleotide polymorphisms (SNPs) are associated with the pathogenesis of diabetes [31,32]. In the current study, PTEN levels were significantly lower in both the DM with microalbuminuria and DM without complications groups compared to the control group. PTEN levels in the DM with microalbuminuria group were also significantly lower than in the DM without complications group. Two major mechanisms underlying the post-translational regulation of PTEN are phosphorylation and oxidation [33]. In the current study, although the mechanism by which high-fasting glucose down-regulates PTEN is not fully determined, miR-223 and miR-106b upregulated in diabetic patients with microalbuminuria. Both miR-223 and miR-106b showed substantial upregulation in the DM with microalbuminuria group, indicating a strong association between miRNA expression and the presence of microalbuminuria in diabetic patients. Also, PTEN and miR-223 were negatively correlated, indicating that they were interrelated in their role within the T1DM pathophysiology. It is likely that upregulation of miR-223 may have reduced the PTEN levels. These results indicate that miR-223 may contribute to the reduction in PTEN that may regulate the pathologic features of microalbuminuria. These results suggest that miR-223 regulates the expression of PTEN in response to high-fasting glucose in T1DM. There are significant differences in HbA1c levels between the groups. Based on the data provided, PTEN can be interpreted as a strong marker of insulin resistance and poor glycemic control in T1DM. Microalbuminuria is usually associated with high HbA1c, so poor glycemic control should be considered in T1DM. Dey et al. [34] reported that, while using renal samples from OVE26 type 1 diabetic mice, they provided evidence for an inverse relationship between the expression of miR-21 and PTEN. In mesangial and proximal tubular epithelial cells, high-glucose-augmented miR-21 targets the PTEN 3′-UTR to reduce the expression of PTEN. The progression and regression of kidney disease each occur commonly after the development of persistent microalbuminuria in T1DM, suggesting that the development of persistent microalbuminuria offers a valuable occasion to assess and target renal interventions. In particular, intensive diabetes therapy appears to improve renal outcomes after the development of persistent microalbuminuria in addition to preventing the initial development of microalbuminuria [35]. The findings of other researchers’ studies have been confirmed by our results [18,34].

miRNA-specific profiles were observed in some body fluids (PBMCs, plasma, serum) from T1DM patients in different studies, and some miRNAs seem to modulate mRNA expressions of the major T1DM autoantigens [12,36,37,38]. miRNA-mediated post-transcriptional inhibition of PTEN to regulate renal function has been documented [18]. In T1DM, hsa-miR-223–3p, one of the upregulated miRNAs with the largest difference in plasma, was selected for in silico analyses [39]. miR-106b was highly expressed in the skeletal muscle of T2DM patients [40]. However, the role of miR-106b in regulating insulin sensitivity and glucose homeostasis remains to be fully elucidated in T1DM. In the current study, the optimal cut-off value for PTEN is approximately 0.259, which indicates that, when PTEN levels fall below this threshold, the likelihood of the individual being diabetic increases significantly (sensitivity: 94%; specificity: 56%). The optimal cut-off value for miR223 is 32.76. At this threshold, miR223 is highly specific, meaning it is very good at correctly identifying control individuals as non-diabetic (sensitivity: 48%; specificity: 98%). Although miR223 and miR106b demonstrated reasonable sensitivity, their lower specificity suggests a higher likelihood of false positives compared to PTEN. In regard to multivariate logistic regression analyses for determining microalbuminuria, using PTEN and miR223 and miR106b together is the most effective model. Overall, PTEN emerged as the most effective biomarker, while miR223 and miR106b may serve as complementary markers in distinguishing diabetic patients with microalbuminuria from those without complications. Many miRNAs are valued as disease markers. We could not demonstrate in the current study that miR-21 acts as a central moderator of the signal transduction pathways involving PTEN, Akt, and TORC1 that contribute to the pathologies of diabetic nephropathy. Targeting miR-21 may be beneficial for patients with diabetic renal dysfunction.

To the best of our knowledge, this is the first demonstration that PTEN and miR-223 were interrelated in their roles within the T1DM pathophysiology. However, this study also has limitations. We evaluated a relatively small number of T1DM cases. Some circulating miRNAs that are consistently dysregulated in T1DM patients were not assessed in the study.

## 5. Conclusions

In conclusion, hyperglycemia is a risk factor for developing microalbuminuria in T1DM. However, among persons who develop microalbuminuria, lower levels of glycemia and the use of intensive insulin therapy remain associated with more favorable long-term renal outcomes. Monitoring blood pressure in addition to microalbuminuria as a follow-up of diabetic nephropathy in patients with T1DM may be necessary and important for early diagnosis. T1DM patients with high microalbumin levels, poor glucose control, and low PTEN levels may require more careful and more frequent monitoring. PTEN expression is another marker with high sensitivity and specificity. Contrary to PTEN, miR223 and miR106b may serve as complementary markers in distinguishing diabetic patients with microalbuminuria from those without complications. Further studies should be performed to clarify the exact mechanisms and possible clinical applications of PTEN, miR223, and miR106b.

## Figures and Tables

**Table 1:** Descriptives and biochemical test results of individuals.

	Controls (n = 50)	DM Without Complications (n = 30)	DM with Microalbuminuria (n = 30)	*p* Value for a	*p* Value for b	*p* Value for c
Age (years)	37.78 ± 8.37	36.37 ± 8.05	39.73 ± 14.39	0.461	0.250	0.250
Gender (F/M)	31/19	15/15	16/14	0.293	0.446	0.796
DM duration (years)	NA	8.2 ± 5.09	8.9 ± 7.33	NA	NA	0.669
Insulin dose (units/kg/day)	-	0.86 ± 0.03	0.89 ± 0.02	NA	NA	0.963
BMI (kg/m²)	24.58 ± 0.8	27.55 ± 3.02	32.38 ± 6.84	<0.001	<0.001	<0.001
Waist Circumference (cm)	93.55 ± 11.23	103.93 ± 9.15	106.6 ± 13.21	<0.001	<0.001	0.373
Systolic Blood Pressure (mmHg)	124.28 ± 6.6	127.33 ± 7.28	138.83 ± 16.33	0.058	<0.001	<0.001
Diastolic Blood Pressure (mmHg)	74.78 ± 4.49	75.4 ± 6.3	81.0 ± 10.29	0.610	<0.001	0.014
Fasting Glucose (mg/dL)	92.78 ± 8.76	152.87 ± 34.65	155.8 ± 85.93	<0.001	<0.001	0.863
HbA1c (%)	5.72 ± 0.16	7.73 ± 0.4	9.39 ± 0.82	<0.001	<0.001	0.314
Insulin (mIU/L)	4.83 ± 1.03	12.45 ± 10.42	18.63 ± 17.52	<0.001	<0.001	0.102
HOMA-IR	1.1 ± 0.24	4.71 ± 2.93	9.17 ± 7.76	<0.001	0.002	0.185
Microalbuminuria (mg/L)	4.95 ± 3.66	4.32 ± 0.29	193.56 ± 247.29	0.355	<0.001	<0.001
Total Cholesterol (mg/dL)	177.5 ± 13.65	202.02 ± 42.79	183.8 ± 46.31	<0.001	0.370	0.119
Triglycerides (mg/dL)	109.62 ± 59.76	195.41 ± 120.63	187.82 ± 176.97	<0.001	0.005	0.847
HDL (mg/dL)	48.06 ± 6.41	46.05 ± 17.31	40.05 ± 9.57	0.460	<0.001	0.102
VLDL (mg/dL)	21.92 ± 11.95	39.08 ± 24.13	37.56 ± 35.39	<0.001	0.005	0.847
LDL (mg/dL)	107.51 ± 16.71	116.89 ± 52.91	106.18 ± 39.89	0.248	0.836	0.380

NA: not applicable; a: control vs. DM without complications; b: control vs. DM with microalbu-minuria; c: DM without complications vs. DM with microalbuminuria.

**Table 2:** Autoantibody data of the patients.

	GADA (Anti-GAD65) (n = 25)	IAA (n = 35)	Islet Cell Antibody (n = 22)
Positive (%)	82.6	52.5	60.3
Negative (%)	17.4	47.5	39.7

GADA: glutamic acid decarboxylase antibody; IAA: autoantibodies against insulin.

**Table 3:** Ratios of PTEN levels to microalbuminuria, HOMA-IR, Insulin, and HbA1c.

	Controls (n = 50)	DM Without Complications (n = 30)	DM with Microalbuminuria (n = 30)	*p* Value for a	*p* Value for b	*p* Value for c
PTEN/Microalbuminuria	0.099 ± 0.076	0.055 ± 0.005	0.003 ± 0.008	<0.001	<0.001	<0.001
PTEN/HbA1c	0.050 ± 0.011	0.030 ± 0.000	0.030 ± 0.007	<0.001	<0.001	1.000
PTEN/Insulin	0.061 ± 0.015	0.029 ± 0.020	0.021 ± 0.017	<0.001	<0.001	0.157
PTEN/HOMA-IR	8.539 ± 6.818	0.070 ± 0.074	2.826 ± 1.907	<0.001	<0.001	0.066

a: means DM without Complications group compared to the control group; b: means DM with microalbuminuria group compared controls; c: means DM with microalbuminuria group compared to DM without complications.

**Table 4:** Logistic regression data for DM with microalbuminuria vs. DM without complications.

Models	P(M)	P(M|data)	BF_M_	BF_10_	R^2^
PTEN > −0.23 + miR223 > 6.19 + miR106b > 154.25	0.250	0.369	1.753	1.000	1.000
PTEN > −0.23	0.083	0.287	4.425	2.333	1.000
PTEN > −0.23 + miR223 > 6.19	0.083	0.172	2.287	1.400	1.000
PTEN > −0.23 + miR106b > 154.25	0.083	0.172	2.287	1.400	1.000
miR106b > 154.25	0.083	1.192 × 10^−22^	1.312 × 10^−21^	9.699 × 10^−22^	0.147
miR223 > 6.19 + miR106b > 154.25	0.083	4.295 × 10^−23^	4.724 × 10^−22^	3.493 × 10^−22^	0.149
miR223 > 6.19	0.083	2.119 × 10^−23^	2.331 × 10^−22^	1.724 × 10^−22^	0.103
Null model	0.250	7.628 ×10^−24^	2.289 × 10^−23^	2.068 × 10^−23^	0.000

## Data Availability

The data underlying this article are available in the article. If needed, please contact the corresponding author. The email address is huzun59@hotmail.com.
